# Rs-198 Liquid Biofertilizers Affect Microbial Community Diversity and Enzyme Activities and Promote *Vitis vinifera* L. Growth

**DOI:** 10.1155/2020/8321462

**Published:** 2020-06-18

**Authors:** Huadan Lu, Zhansheng Wu, Wenfei Wang, Xiaolin Xu, Xiaochen Liu

**Affiliations:** ^1^School of Chemistry and Chemical Engineering, Shihezi University, Shihezi 832003, China; ^2^School of Environmental and Chemical Engineering, Xi'an Polytechnic University, Xi'an 710048, China

## Abstract

Chemical fertilizers were applied on perennial tree vines to obtain high yields, which have resulted in considerable deterioration of soil quality, and it is likely to have negative impacts on the development of the grape industry. In this study, *P. putida* Rs-198 liquid biofertilizer (Rs198LBF) was inoculated into grape rhizosphere soils to assess its influence on grape growth and microbial community. Field experiment results showed that grape growth and quality increased depending on the concentrations of Rs198LBF applications. The berry weight, length, and width in addition with 60 ml Rs198LBF (1.44 × 10^13^ cfu ml^−1^ Rs-198) per grapevine treatment (BFP3) were 17.2%, 6.2%, and 4.4% higher than those of CK (control, non-inoculation) treatment, respectively. The available phosphorus contents in addition with 40 ml Rs198LBF per grapevine (BFP2) and BFP3 treatments were 12.6% and 55.3% higher than those of CK treatment (*P* < 0.05). The activities of invertase and alkaline phosphatase were improved in BFP2 and BFP3 treatment compared with those in CK. The relative abundance of potentially beneficial bacteria significantly increased compared with that in CK treatment (*P* < 0.05). The clusters of orthologous groups (COG) annotation illustrated that the application of 60 ml Rs198LBF increased the relative abundance of metabolic genes in rhizosphere soil. The results of this study show that biofertilizer is very effective in enhancing plant growth and affects soil community diversity.

## 1. Introduction

Red Globe grape (*Vitis vinifera* L.) is an economically important fruit crop in the Xinjiang province of China. Xinjiang province is located in the north at latitudes of 30-45 degrees, which is the golden area for growing grapes in the world [1]. For many years, chemical fertilizers were applied on perennial tree vines to obtain high yields, which have resulted in considerable soil quality deterioration, loss of productivity, and large-scale ecosystem degradation in the long term [2, 3]. However, the utilization of microorganisms is useful in the development of sustainable agriculture and to minimize the use of inorganic chemicals. Biofertilizers are effective alternative to chemical fertilizers to improve sustainable agriculture soil fertility and crop yield which can preserve the environment in the long run [4, 5].

Plant growth-promoting rhizobacteria (PGPR) are commonly used to prepare biofertilizer, which can promote plant growth by solving phosphorus, fixing nitrogen, releasing potassium, or producing hormone production [6, 7]. Biofertilizers have a positive effect on soil physical and chemical characteristics [8] and consequently promote plant growth and develop fruit production and quality [8]. Shiraz grape yield was 13.9% higher and Brix was 3.04% higher in the plantation where the biofertilizer was applied than CK treatment [9]. In addition, nutrient cycling and organic matter turnover in terrestrial ecosystem were influenced by the soil microorganisms [10]. Yu et al. investigated the changes in Pb-contained soil on enzyme activities and found that *Pseudomonas* sp. GHD-4 inoculation could stimulate sucrase and polyphenol oxidase activities [11]. Moreover, the application of biofertilizer increased vegetative growth by enhancing plant height, leaf number, and total dry weight of tomato plant under field conditions [5]. Biofertilizers made from rocks and elemental sulphur inoculated with *Acidithiobacillus* improve the yield of many short-cycle grape plants similarly to soluble fertilizers [12]. *P. putida* Rs-198 was isolated in our laboratory and demonstrated a significant ability to promote the growth of plant [13]. Yao et al. reported that Rs-198 could release salt stress by increasing cotton plants' uptake of Mg^2+^, K^+^, and Ca^2+^ in soil and decreasing their uptake of the Na^+^. Moreover, Rs-198 promoted the content of indole acetic acid (IAA) by cotton [13]. In addition, He et al. reported that the application of *P. putida* Rs-198 was effective in combating salinity [14]. Under salt stress conditions, the Rs-198 biofertilizers remarkably increased soluble protein content and reduced malonaldehyde (MDA) and proline accumulation in cotton seedlings [2].

Previous studies of our laboratory focused on the effects of *P. putida* Rs-198 liquid biofertilizers (Rs198LBFs) on cotton in the laboratory, but had not been used on the field scale. Some researchers proved that laboratory results could provide guidance and reference for field applications [15]. It was not clear if Rs198LBF could affect grape growth and change soil microbial community diversity and enzyme activities. Stamford et al. showed that biofertilizers influenced positively on total N, available P, available K, exchangeable Ca^2+^ and Mg^2+^ in soil and also increased the grape yield as compared to the control when applied at a depth of 0-20 cm [12]. Soil microbial communities perform key functions of maintaining soil productivity [16]. Some researchers have shown that soil microbial community diversity was obviously affected due to the use of biofertilizers [17]. Dong et al. revealed that biofertilizers promoted the relative abundance of potentially beneficial bacterial taxa (*Bacillus*, *Burkholderia*, *Rhizobium*, *Streptomyces*, and *Mycobacterium*) and increased the yield of *P. ginseng* as well [18]. Therefore, it is essential to understand how Rs198LBF influences the growth of grapevine and soil microbial diversity for its agricultural field application.

In the present study, we conducted a one-season field experiment in perennial vine treated with Rs198LBF of different concentrations to analyze the responses of the rhizosphere microbial communities by sequencing the bacterial communities. The research objectives include the following: (1) to explore the effects of different amounts of Rs198LBF on grape growth; (2) to evaluate the impact of different amounts of Rs198LBF on rhizosphere soil physicochemical properties and enzyme activities; and (3) to determine changes of Rs198LBF on the microbial community diversity annually in grapevine rhizosphere soil.

## 2. Materials and Methods

### 2.1. Field Site

The field trial was carried out at the Standard Grape Demonstration Park in the Experimental Station of the Agricultural College of Shihezi University, Xinjiang Province, China (N44°26′, E85°95′; elevation 427 m). The annual average temperature is 8.5°C. The soil type is grey desert soil. Prior to treatment, the basic soil properties were as follows: pH 8.12 (5 : 1 water to soil ratio), EC 154.9 *μ*s cm^−1^, total nitrogen 0.89 g kg^−1^, total phosphorus 1.06 g kg^−1^, total potassium 22.58 g kg^−1^, and organic matter (OM) 13.27 g kg^−1^.

### 2.2. Preparation of Biofertilizer

*P. putida* Rs-198 was isolated in our laboratory [13]. The NA liquid medium was used for *P. putida* Rs-198 pure cultures. Subsequently, the bacteria were cultured at 30°C and at 170 rpm for 36 h. Then, the cell concentration in the broth was measured, which was up to 1.8 × 10^13^ cfu ml^−1^. The *P. putida* Rs-198 suspensions were then used to prepare *P. putida* Rs-198 liquid biofertilizer (Rs198LBF). Rs198LBF was prepared following the liquid formulation of He et al. [2]. Briefly, Rs198LBF contains 30 g humic acid, 20 g urea, 50 g corn flour, 10 g bentonite, 20 g alginate, 20 g KCl, 30 g K_2_SO_4_, 40 g (NH_4_)_2_HPO_4_, and 1000 ml H_2_O, and the *P. putida* Rs-198 suspension content is 80% (wt. %). The effective number of Rs198LBF was 1.44 × 10^13^ cfu ml^−1^. The solid matter particle size was less than 0.1 mm.

### 2.3. Field Experimental Design and Soil Sampling

This study was performed in a stochastic design, and each treatment was repeated three times. The base fertilizer is chemical fertilizer, and NPK application rates were 108 kg N ha^−1^, 60 kg P_2_O_5_ ha^−1^, and 130 kg K_2_O ha^−1^, respectively. Four treatments were designed as follows: CK treatment (noninoculated control), BFP1 treatment (addition with 20 ml containing 28.8 × 1013 cfu Rs-198 Rs198LBF per grapevine), BFP2 treatment (addition with 40 ml containing 57.6 × 1013 cfu Rs-198 Rs198LBF per grapevine), and BFP3 treatment (addition with 60 ml containing 86.4 × 1013 cfu Rs-198 Rs198LBF per grapevine). The different amounts of Rs198LBFs diluted ten times using distilled water were irrigated equally four times into the ditch around (about 10 cm from the main stem) the vine during the grape germination, flowering, fruiting, and ripening periods, respectively. The same amount of water in CK treatment was irrigated during each period.

During the harvest stage, six grapevines were selected from each treatment, and thirty fruits were picked from each grapevine. All sampled fruits were immediately loaded into plastic boxes and then moved to the laboratory for the grape size determination. The width and length of each grape were measured using a vernier caliper. The selected grapes of each treatment were weighed to stand for the average berry weight. Fruit shape index is the ratio of fruit length to fruit width [19]. After the grapes were picked, the rhizosphere soil of six grape trees randomly selected was taken at a depth of 0-20 cm for each treatment. The fresh soils were mixed to form one composite sample. Plant residues and stones in the rhizospheric soil were removed by 2 mm mesh. Then, soil samples were divided into two parts: one part was air-dried for soil enzyme activity and physicochemical property analysis, while the other part was stored in sealed sterile cryogenic vials at -80°C for DNA extraction.

### 2.4. Soil Physicochemical Property Analysis

The pH of the soil was measured in soil : water suspension (1 : 5) using a pH meter (PHS-3C, Shanghai Leici Instrument, China). Soil OM (organic matter), alkaline nitrogen (AN), available phosphorus (AP), and available potassium (AK) concentrations were determined as described previously [20].

### 2.5. Soil Enzyme Activity

In order to measure the effect of Rs198LBF application on soil fertility, the activities of the three enzymes were measured in air-dried soil extracts. Alkaline phosphatase activity in the soil was measured as described before [21]. The soil urease was determined by sodium phenolate-sodium hypochlorite colorimetry [22]. Invertase activity in soil was determined by the 3,5-dinitrosalicylic acid (DNS) method described by Frankeberger and Johanson [23].

### 2.6. Bacterial 16S rRNA Gene PCR and High-Throughput Sequencing

Power soil DNA isolation kit (MO BIO Laboratories) was used for DNA (soil sample) extraction. The quality of DNA was evaluated by the ratios of 260 nm/280 nm and 260 nm/230 nm. The V3-V4 region of 16S rRNA genes was used to evaluate soil bacterial diversity and community, respectively. The hypervariable region V3-V4 of the bacterial 16S rRNA gene was amplified using the universal primers (338F: 5′-ACTCCTACGGGAGGCAGCA-3′ and 806R: 5′-GGACTACHVGGGTWTCTAAT-3′ synthesized at Sangon Inc., China). The PCR amplification was performed in a total volume of 50 *μ*l, which was composed of 10 *μ*l buffer, 0.2 *μ*l Q5 high-fidelity DNA polymerase, 10 *μ*l high GC enhancer, 1 *μ*l dNTP, 10 *μ*M each primer, and 60 ng genome DNA. The PCR amplification procedure was 98°C for 2 min, with 30 cycles of 30 s at 98°C, 30 s at 50°C, and 30 s at 72°C, and finally extended at 72°C for 5 min. After amplification, the products were purified through VAHTSTM DNA Clean Beads. Then, the second round of PCR was performed in a 40 *μ*l reaction which contained 20 *μ*l 2x Ph*μ*sion HF MM, 8 *μ*l ddH_2_O, 10 *μ*M of each primer, and 10 *μ*l PCR products from the first step. Reaction products were then pooled and quantified with a NanoDrop 2000 spectrophotometer (Thermo Scientific, USA). High-throughput sequencing analysis of 16S rRNA genes was performed on the purified, pooled sample using the Illumina HiSeq 2500 platform.

### 2.7. Optimize High-Throughput Sequencing Data

After the sequencing is completed, the FLASH v1.2.11 software was used to splice the reads of each sample by overlap, and the obtained splicing sequence was then filtered using the Trimmomatic v0.33 software to obtain high-quality tag data. Finally, the UCHIME v8.1 software was used to recognize and remove the chimeric sequences to acquire the final valid data. Reads with a length ≥ 400 bp were kept for the following analysis. The clusters were clustered at 97% similarity level using USEARCH v10.0 in QIIME (version 1.8.0) software. Operational taxonomic units (OTU) was obtained, and the OTU was taxonomically annotated based on the Silva (bacteria) taxonomic database. Rarefaction curve was analyzed by Mothur v.1.30 (http://www.mothur.org/). Principal coordinate analysis (PCoA) was performed to compare groups of samples according to Bray-Curtis distance metrics. COG function information for 16S copy number was predicted by corresponding to the Greengenes release version gg_13_5 (http://greengenes.secondgenome.com/). PICRUSt v1.0.0 was used to analyze the clusters of orthologous groups (COG) function.

### 2.8. Data Calculation and Statistical Analysis

All soil physicochemical property data and enzymatic activity data were analyzed using Origin 9.0 and Excel 2010. Grape growth and soil physicochemical and enzymatic activities were analyzed by one-way analysis of variance (ANOVA) and Duncan's multiple comparisons (*P* < 0.05). All assays were performed in triplicates.

## 3. Results

### 3.1. Grape Growth

The grape growth and quality of grapevine increased depending on the concentrations of Rs198LBF application (Table 1). Treatments by different concentrations of Rs198LBF all promoted the growth of grapes. BFP3 was the best. BFP2 and BFP3 significantly increased grape weight, grape length, and grape width, respectively, as compared to those without biofertilizers. The berry weight in BFP2 and BFP3 treatments was all 17.2% higher than that in CK treatment (*P* < 0.05). The berry length in BFP2 and BFP3 treatments was 5.5% and 6.2% higher than that in CK treatment, respectively (*P* < 0.05). The berry width in BFP2 and BFP3 treatments was 3.6% and 4.4% higher than that in CK treatment (*P* < 0.05). It was observed that the use of different concentration of Rs198LBF could not change the shape index of grape, and the fruit shape index of grapes was all about 1.10. Moreover, for grape quality, it was observed that the fruit soluble solid was directly proportional to the amount of biofertilizer used, and fruit hardness was inversely proportional to the amount of biofertilizer used.

### 3.2. Soil Physicochemical Properties

The changes of different concentrations of Rs198LBFs on rhizosphere soil physicochemical characteristics were shown in Table 2. Our results showed that several physicochemical and biochemical properties of the rhizosphere soil were changed in response to the application of Rs198LBF. AN and OM of soils treated with Rs198LBF showed insignificant differences compared with CK treatment. However, the contents of AK and AP increased in all treatments. Soil AP was changed insignificantly in BFP1 treatment, while the AP contents in BFP2 and BFP3 treatment were 12.6% and 55.3% higher than those in CK treatment, respectively (*P* < 0.05). In addition, we observed more acid pH values in the treatment with higher bacterial density. The content of AK in the soil was from high to low as BFP2, BFP3, BFP1, and CK, respectively. The AK content in BFP2 and BFP3 was 87.9% and 47.0% higher than that in CK, respectively (*P* < 0.05). Overall, the application of Rs198LBF improved greatly the soil properties and fertility.

### 3.3. Soil Enzyme Activity

**Figure 1:**
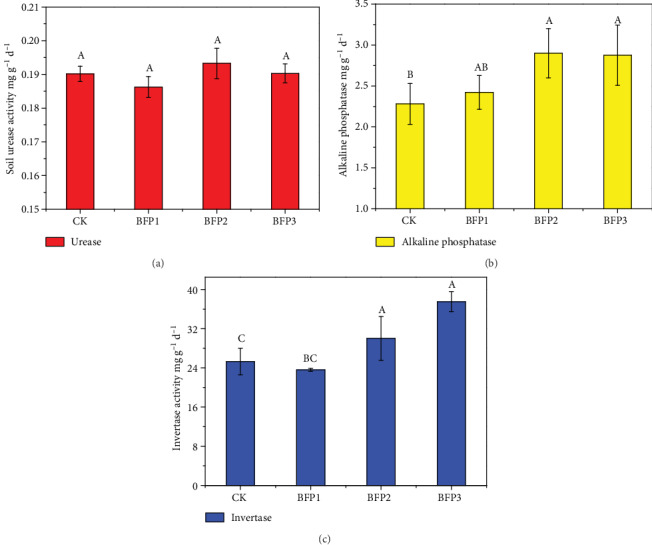
Effect of different biofertilizer treatments on soil enzyme activities: (a) urease activity; (b) alkaline phosphatase activity; (c) invertase activity. Values are reported as repeated mean ± standard error (*P* < 0.05). According to Duncan's test, the different letters (such as a, b) on the graph bars represent significant difference at *P* < 0.05.

After grape harvesting, the enzyme activities were determined in the soils treated with CK and Rs198LBFs. The influences of different concentrations of Rs198LBFs on rhizosphere soil enzyme activities are described in Figure 1. Urease activity was influenced insignificantly after Rs198LBF addition (Figure 1(a)). But alkaline phosphatase activity of soil was positively affected in the treatment with Rs198LBFs. When compared with the CK treatment, the alkaline phosphatase activity of soil in the BFP2 and BFP3 treatments was significantly increased by 26.11% and 27.15% (*P* < 0.05), respectively (Figure 1(b)). Similar to the activity of alkaline phosphatase, invertase activity was actively affected by the different concentrations of Rs198LBFs (Figure 1(c)). BFP2 and BFP3 treatments significantly increased invertase activity of soil by 18.67% and 48.33% (*P* < 0.05), respectively, as compared with the CK treatment (Figure 1(c)).

### 3.4. General Analyses of the High-Throughput Sequencing Results

**Figure 2:**
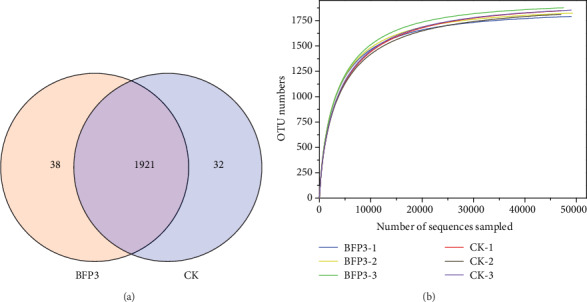
(a) Venn diagram of the two samples. Different colors represent different treatments. Blue: BFP3; pink: CK; purple: CK and BFP3. The mean OTU value of three biological replicates of each treatment was used in the analysis. (b) Rarefaction curves of differently treated soil samples at 0.97 levels.

**Figure 3:**
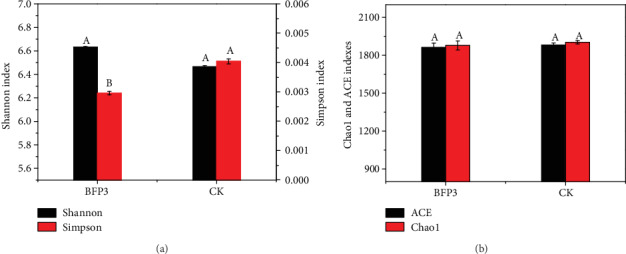
Alpha diversity of bacterial community in the two samples. (a) Shannon and Simpson indexes. (b) Chao1 and ACE indexes. According to Duncan's test, the different letters (such as a, b) on the graph bars represent significant difference at *P* < 0.05.

In order to evaluate the changes of Rs198LBFs on microbial community diversity, the microbial diversity was determined in CK and BFP3 treatment soils. A total of 375,918 effective 16S rDNA sequences (clean date) and 1991 OTUs were obtained (Table 3). The OTUs ranged from 1792 to 1877 in the six samples. 1921 OTUs were common among the two treatments. 32 and 38 unique OTUs were observed in the CK and BFP3 treatments, respectively, in the Venn diagram (Figure 2(a)). Under experimental conditions, the number of sequences of the soil samples in CK and BFP3 treatments was increased to 40000 (Figure 2). The curve tends to be flat, indicating that the Illumina MiSeq sequencing of this study has obtained most of the bacterial sequences in soil samples, which could reflect the bacterial community composition of rhizosphere soil. It is interesting to note that no differences were observed between CK treatment and BFP3 treatment using Shannon, ACE, and Chao1 richness estimators (*P* > 0.05) (Figure 3), while Simpson was significantly lower in BFP3 soils than in CK soils (Figure 3(a), Table S5).

### 3.5. Rhizosphere Soil Bacterial Community Diversity

**Figure 4:**
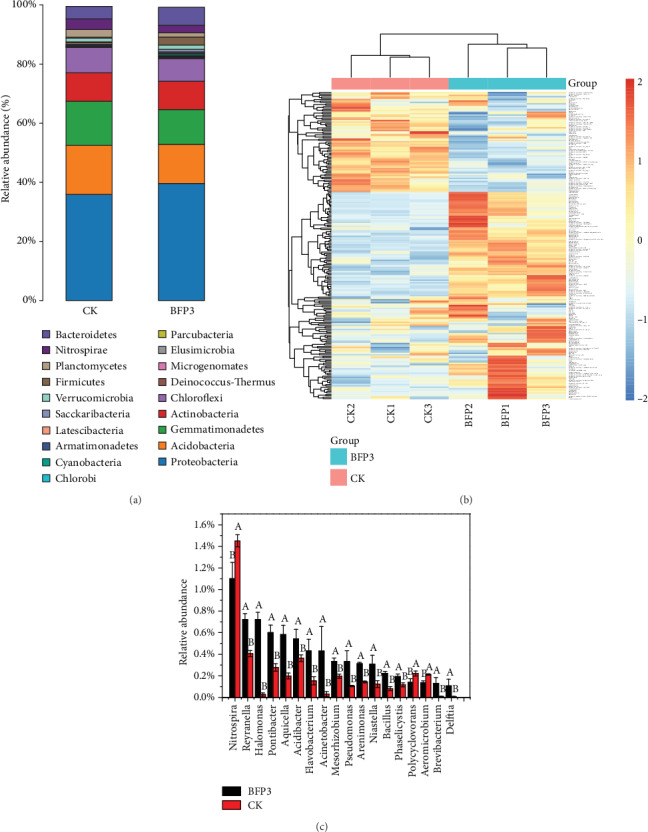
(a) Relative abundance of top 0.1% percent phyla in the rhizosphere. (b) Relative abundance (>0.1%) of bacterial groups in grapevine rhizosphere of CK and BFP3 soils at the family level. (c) Changes in bacterial genera in CK and BFP3 soils. Samples for each group are represented by three replicates. According to Duncan's test, the different letters (such as a, b) on the graph bars represent significant difference at *P* < 0.05.

The relative abundance of bacterial groups changed in grapevine rhizosphere soils from the phylum level to the genus level (Figure 4, Tables S1–S3). The dominant bacterial taxa were *Proteobacteria*, *Acidobacteria*, *Gemmatimonadetes*, *Actinobacteria*, *Chloroflexi*, and *Bacteroidetes* at the phylum level in rhizospheric soils of grapevine (Figure 4(a), Table S1). The highest proportion phylum in the two samples was *Proteobacteria* (Table S1). *Firmicutes*, *Bacteroidetes*, *Saccharibacteria*, *Cyanobacteria*, *Deinococcus-Thermus*, and *Spirochaetae* abundance significantly increased in BFP3 treatment as compared in CK treatment (*P* < 0.05). Moreover, the relative abundance of *Acidobacteria*, *Planctomycetes*, *Gemmatimonadetes*, and *Nitrospirae* showed significantly decreasing trends in BFP3 soils than in CK soils (*P* < 0.05), while the relative abundance of *Actinobacteria*, *Armatimonadetes*, *Chlorobi*, *Parcubacteria*, *Latescibacteria*, *Elusimicrobia*, *Microgenomates*, *Verrucomicrobia*, *Chloroflexi*, and *Proteobacteria* in BFP3 soils showed insignificant differences as compared with that in CK soils (*P* > 0.05). At the family level, the relative abundance of bacterial groups revealed clearly changed between the two treatments (Figure 4(b), Table S2).

Furthermore, the genus relative abundances (%) of *Bacillus*, *Halomonas*, *Delftia*, *Brevibacterium*, *Acinetobacter*, *Pseudomonas*, *Aquicella*, *Flavobacterium*, *Niastella*, *Arenimonas*, *Pontibacter*, *Reyranella*, *Mesorhizobium*, *Phaselicystis*, and *Acidibacter* in BFP3 soils were significantly increased (*P* < 0.05), while the genus relative abundances (%) of *Nitrospira*, *Aeromicrobium*, and *Polycyclovorans* in BFP3 soils declined compared with those in CK-treated soils (*P* < 0.05) (Figure 4(c), Table S3).

### 3.6. Beta Diversity

**Figure 5:**
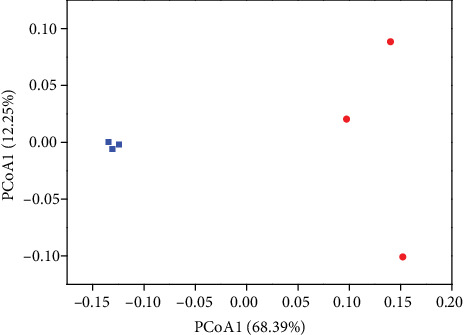
PCoA of sequencing data of bacteria in CK and BFP3 soils (red: BFP3; blue: CK).

In order to define the changes in the community structure of rhizosphere soil between CK and BFP3 soils, the *β* diversity index of the soil bacterial community was calculated. The changes of the bacterial communities in BFP3 soils were showed by PCoA ordination compared to those in CK soils (Figure 5). The first principal component axis (68.39% contribution) revealed that bacterial communities in BFP3 soils differed from those in CK soil (Figure 5).

### 3.7. COG Function Analysis

**Figure 6:**
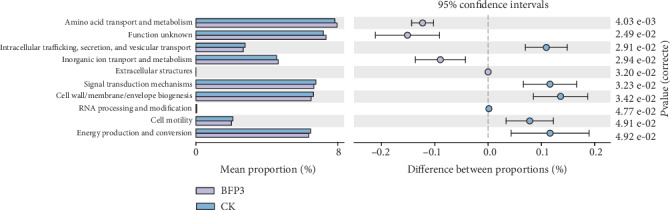
Analysis of COG function differences between CK and BFP3 soils. The left part of the figure shows the abundance ratio of different functions in the two groups of samples, the middle part shows the difference ratio of the function abundance in the 95% confidence interval, and the rightmost value is the *P* value.

At level 2 of COG, a total of 10 different categories were predicted in all treatments (Figure 6, Table S4). Amino acid transport and metabolism, energy production and conversion, cell wall/membrane/envelope biogenesis, signal transduction mechanisms, and function unknown were the dominant functions among the 10 categories. Inorganic ion transport and metabolism and amino acid transport and metabolism were observed to be higher in BFP3 treatment soils than in CK soils (Figure 6, Table S4).

## 4. Discussion

The key objective of this research was to define if Rs198LBF inoculation could improve the microbial community diversity of the vineyard and improve the quality of the grapes. Biofertilizers are widely accepted to replace chemical fertilizers because they promote plant growth and soil fertility [24]. The results of biofertilizer application may offer information about improving fruit quality [5, 7, 9]. Biofertilizers which contained *Azotobacter* sp., *Azospirillum* sp., and *Pseudomonas sp*. with peat could increase vegetative growth and plant production of tomato [5]. Kok et al. showed that foliar microbial fertilizer applications contributed to the increase in berry width, length, and weight and decreased content of titratable acid compared to CK treatment [25]. Ju et al. reported that biofertilizers, including plant growth-promoting rhizobacteria, *Pseudomonas*, significantly increased the availability of nutrients and improved crop yield [24]. The use of Rs198LBFs increased grape weight, grape length, and grape width, respectively, as compared to CK treatment (Table 1). Liu et al. showed that the inoculation with phosphate-solubilizing bacteria significantly facilitated the plant height, stem thickness, and root and shoot dry weight and increased the available P content in soils compared to CK treatment [1]. Our previous research showed that *Pseudomonas* can significantly increase the fresh weight and dry weight of cotton [2]. Moreover, soil AP and AK contents in the grapevine rhizosphere were improved and correlated with Rs198LBF concentration, but the use of Rs198LBFs did not influence soil AN and OM contents in the grapevine rhizosphere (Table 2). Significantly higher available P in soil may be related to organic acid released by plant roots in the rhizospheric soil [13]. Our laboratory previous results also revealed that Rs-198 has a good ability on phosphate solubilization and increases nutrient availability of soil [26]. Otherwise, findings from Li et al. similarly demonstrated that *Bacillus cereus* Pb25 can increase soil available phosphorus [27]. In addition, soil enzyme activities are the indicator of ecosystem health and sustainability [28]. Invertase, urease, and phosphomonoesterase activities are closely connected with the C, N, and P looping in soil [29]. In the present study, the application of Rs198LBF has an obvious increase in soil invertase and alkaline phosphatase activity as compared with CK treatment (Figure 1), indicating that Rs198LBF application could boost soil enzyme activities. The increase in the enzymatic activities may be related to changes in rhizosphere bacteria activity [30]. Application of biofertilizer may change native microbial diversity [18, 31].

Soil microbial communities were considered as sensitive indicators of the remediation process, as the population, structure, and even diversity of the community are susceptible to years of farming. Grapes are perennial vines that cannot be rotated, fertilization and irrigation methods are limited, and the soil microbial diversity is significantly affected [32]. PGPR successfully colonized rhizosphere soils, which promoted the yield of orange [33]. After the application of biofertilizer which contained PGPR, potentially beneficial bacterial groups (*Bacillus*, *Burkholderia*, *Rhizobium*, *Streptomyces*, and *Mycobacterium*) revealed adding trends [18]. Early researches in our laboratory confirmed that Rs-198 changed pepper rhizospheric bacterial diversity after inoculation [14]. In the present research, *Firmicutes*, *Bacteroidetes*, *Saccharibacteria*, *Cyanobacteria*, *Deinococcus-Thermus*, and *Spirochaetae* abundance obviously increased in BFP3 treatment soils in contrast with CK treatment soils (Figure 4(a) and Table S1). Most of these phyla had actively influenced soil remediation and plant growth. Starr et al. predicted that *Saccharibacteria* generates energy by resolving cellulose, hemicellulose, and 1,3-beta-glucan and consequently promotes plant growth [34]. *Cyanobacteria* promoted plant growth and relieved biotic and abiotic stress [35]. In our previous research, it was also found that *Cyanobacteria* were enhanced in rhizosphere soil than bulk soil of pepper after the application of Rs-198 [14].

Furthermore, at the genus level, the use of Rs198LBF increased the abundance of potentially beneficial bacteria such as *Halomonas*, *Delftia*, *Brevibacterium*, *Acinetobacter*, *Pseudomonas*, and *Bacillus* (Figure 4(b) and Table S3). This was consistent with previous reports which demonstrated that biofertilizer containing PGPR can increase the abundance of potentially beneficial bacteria [31]. The relative abundance of *Bacillus* significantly increased after the application of biofertilizer as compared with control [18]. *Bacillus* promoted the yield of cowpea and may perform the role by further altering the composition of leaf microbiota [36]. The high amount of *Pseudomonas* may be related to colonization of *P. putida* Rs-198. Researches indicated that the changes in microbial diversity may be related to the fertilizer application, and the application of biofertilizer can maintain the balance of microbial diversity [18, 27]. In this research, the reason for the increase in vineyard soil microbial diversity may be related to the application of Rs198LBF. It was speculated that the application of biofertilizers promotes grape growth by increasing the amount of soil available phosphorus, modifying the soil communities, and affecting soil enzymes. Compared to CK, inorganic ion transport and metabolism and amino acid transport and metabolism were observed to be higher in BFP3 treatment soils, which indicated that BFP3 treatment promoted the metabolism of the microorganisms. He reported that glycan biosynthesis and metabolism and enzyme families' energy metabolism were higher in the Rs-198 inoculated soil than in CK soil [14]. The results of this study are guided for the application of Rs198LBF in viticulture.

In conclusion, the results of this study showed that the application of Rs198LBF promoted alkaline phosphatase and invertase activity, increased the amount of available phosphorus, and enhanced the growth and quality of grape. Moreover, BFP3 treatment affected the soil bacterial community diversity. The abundances of potentially beneficial bacteria were increased, and these were potential promotion of grape growth in BFP3 treatment. Furthermore, the application of Rs198LBF increases the inorganic ion transport and metabolism and amino acid transport and metabolism of the microorganisms. These results provide material facts for the application of biofertilizer and provide a reference for sustainable development.

## Figures and Tables

**Table 1:** Effects of different biofertilizer treatments on the appearance and quality of berries.

Treatment	Berry weight (g)	Berry length (mm)	Berry width (mm)	Shape index	Total soluble solid (%)	Hardness
CK	9.05 ± 0.49^b^	25.66 ± 1.13^c^	23.88 ± 0.82^b^	1.08 ± 0.06^a^	16.55 ± 0.51^b^	2.25 ± 0.09^a^
BFP1	9.58 ± 0.82^b^	26.34 ± 0.71^bc^	24.43 ± 0.91^ab^	1.08 ± 0.03^a^	16.66 ± 0.81^ab^	2.25 ± 0.23^a^
BFP2	10.28 ± 0.43^a^	27.08 ± 1.08^ab^	24.75 ± 0.68^a^	1.09 ± 0.06^a^	16.69 ± 0.85^ab^	1.93 ± 0.19^ab^
BFP3	10.61 ± 0.91^a^	27.24 ± 0.94^a^	24.92 ± 0.83^a^	1.09 ± 0.05^a^	17.70 ± 0.84^a^	1.88 ± 0.25^b^

Values are reported as repeated mean ± standard error. According to Duncan's test, the different letters (such as a, b, and c) in each column represent significant difference at *P* < 0.05.

**Table 2:** Effect of different biofertilizer treatments on soil physicochemical properties.

Treatments	pH	AN (mg kg^−1^)	AP (mg kg^−1^)	AK (mg kg^−1^)	OM (g kg^−1^)
CK	7.93 ± 0.15^a^	34.68 ± 1.05^a^	18.65 ± 1.67^b^	130.45 ± 3.95^d^	14.24 ± 1.43^a^
BFP1	7.70 ± 0.20^ab^	40.69 ± 4.19^a^	18.73 ± 0.62^b^	173.94 ± 17.48^c^	13.09 ± 0.82^a^
BFP2	7.59 ± 0.13^b^	40.00 ± 2.08^a^	21.00 ± 3.62^ab^	245.11 ± 24.32^a^	14.17 ± 1.93^a^
BFP3	7.49 ± 0.02^b^	44.00 ± 3.22^a^	28.96 ± 1.05^a^	191.74 ± 19.78^b^	15.57 ± 0.78^a^

Values are reported as repeated mean ± standard error. According to Duncan's test, the average of the different letters (such as a, b, and c) in each column was significantly different at *P* < 0.05.

**Table 3:** OTUs and clean date of bacterial sequences in each sample.

Treatments	OTUs	Clean date
CK-1	1847	63057
CK-2	1813	62290
CK-3	1854	63791
BFP3-1	1792	62967
BFP3-2	1824	63408
BFP3-3	1877	60405

-1, -2, and -3 presented three replicates. Clean date was effective 16S rDNA sequences.

## Data Availability

The data used to support the findings of this study are available from the corresponding author upon request.

## Supplementary Materials

Supplementary Materials Table S1: ANOVA for phylum abundance. Table S2: ANOVA for family abundance. Table S3: ANOVA for genus abundance. Table S4: Analysis of COG function differences between in CK and BFP3 soils. Table S5: Alpha diversity of bacterial community in the two samples.
