# Supplementary material for: Rs-198 Liquid Biofertilizers Affect Microbial Community Diversity and Enzyme Activities and Promote Vitis vinifera L. Growth
Source: Biomed Res Int. 2020 Jun 18;2020:8321462. doi: 10.1155/2020/8321462 (PMC7321499; doi:10.1155/2020/8321462)
Supplement: Supplementary Materials — Table S1: ANOVA for phylum abundance. Table S2: ANOVA for family abundance. Table S3: ANOVA for genus abundance. Table S4: Analysis of COG function differences between in CK and BFP3 soils. Table S5: Alpha diversity of bacterial community in the two samples. [file 8321462.f1.docx]

**Table S1** ANOVA for phylum abundance

| phylum | Relative abundance (%) (Mean±SE) | | P-value |
| --- | --- | --- | --- |
|  | BFP3 | CK |  |
| *Proteobacteria* | 39.5940±1.7538a | 35.923±0.5681a | 0.053924 |
| *Acidobacteria* | 13.2373±0.5639b | 16.6081±0.3559a | 0.010271 |
| *Gemmatimonadetes* | 11.8052±1.0327b | 14.9065±0.4839a | 0.030447 |
| *Actinobacteria* | 9.5527±0.5271a | 9.6156±0.0827a | 0.903438 |
| *Chloroflexi* | 7.7262±0.6677a | 8.6591±0.0756a | 0.135534 |
| *Bacteroidetes* | 6.1472±0.3801a | 4.2696±0.2777b | 0.012674 |
| *Firmicutes* | 2.6612±1.0116a | 0.3117±0.1245b | 0.04121 |
| *Nitrospirae* | 2.4494±0.2065b | 3.5636±0.1224a | 0.00856 |
| *Planctomycetes* | 1.4883±0.3018b | 2.6309±0.1262a | 0.015521 |
| *Verrucomicrobia* | 1.4635±0.1745a | 1.2461±0.084a | 0.214708 |
| *Saccharibacteria* | 0.8887±0.0741a | 0.3433±0.0808b | 0.008219 |
| *Cyanobacteria* | 0.5499±0.1467a | 0.0472±0.0106b | 0.014948 |
| *Latescibacteria* | 0.3848±0.0927a | 0.4459±0.0299a | 0.485329 |
| *Armatimonadetes* | 0.3696±0.0339a | 0.3745±0.0287a | 0.882884 |
| *Deinococcus-Thermus* | 0.2166±0.0238a | 0.0433±0.0102b | 0.010501 |
| *Microgenomates* | 0.2070±0.1312a | 0.0675±0.0071a | 0.230534 |
| *Chlorobi* | 0.1903±0.0609a | 0.1756±0.016a | 0.776626 |
| *Parcubacteria* | 0.1868±0.0413a | 0.1588±0.0224a | 0.475614 |
| *Spirochaetae* | 0.1493±0.0575a | 0.0172±0.0133b | 0.042161 |
| *Elusimicrobia* | 0.1428±0.0462a | 0.1735±0.0111a | 0.488404 |

**Table S2** ANOVA for Family abundance

| Family | Relative abundance (%) (Mean±SE) | | P-value |
| --- | --- | --- | --- |
|  | BFP3 | CK |  |
| *Gemmatimonadaceae* | 9.1257±0.7767b | 12.0015±0.4136a | 0.004801 |
| *Sphingomonadaceae* | 5.0758±0.3791b | 5.8899±0.2989a | 0.043199 |
| *Nitrosomonadaceae* | 3.2275±0.1603b | 4.7946±0.2101a | 0.000507 |
| *Erythrobacteraceae* | 1.1475±0.0127a | 0.9603±0.0734b | 0.012143 |
| *Nitrospiraceae* | 1.0532±0.1466b | 1.3959±0.0561a | 0.019393 |
| *Rhodobacteraceae* | 0.8537±0.1619a | 0.4893±0.0709b | 0.023381 |
| *Longimicrobiaceae* | 0.8491±0.0737b | 1.0359±0.0380a | 0.017532 |
| *Comamonadaceae* | 0.8042±0.2112a | 0.3404±0.0628b | 0.021835 |
| *Flavobacteriaceae* | 0.7797±0.2156a | 0.2052±0.0465b | 0.010721 |
| *Sandaracinaceae* | 0.7563±0.1372a | 0.4374±0.0516b | 0.019643 |
| *Hyphomicrobiaceae* | 0.7375±0.0422a | 0.6034±0.0089b | 0.005766 |
| *Phycisphaeraceae* | 0.7350±0.1830b | 1.3749±0.1577a | 0.010124 |
| *Coxiellaceae* | 0.5587±0.0801a | 0.2079±0.0216b | 0.001849 |
| *Iamiaceae* | 0.5475±0.0161b | 0.7965±0.0615a | 0.002461 |
| *Gaiellaceae* | 0.5197±0.0103a | 0.4330±0.0270b | 0.006541 |
| *Bradyrhizobiaceae* | 0.5097±0.0192a | 0.2623±0.0187b | 0.00009 |
| *Verrucomicrobiaceae* | 0.4175±0.1240a | 0.1570±0.0382b | 0.025434 |
| *Solirubrobacteraceae* | 0.3352±0.0202a | 0.2873±0.0190b | 0.040614 |
| *Pseudomonadaceae* | 0.3214±0.0964a | 0.1056±0.0044b | 0.01792 |
| *Phyllobacteriaceae* | 0.3196±0.0300a | 0.189±0.0187b | 0.003068 |
| *Microbacteriaceae* | 0.3119±0.0433a | 0.2193±0.0303b | 0.038635 |
| *Oxalobacteraceae* | 0.2663±0.0503a | 0.1116±0.0482b | 0.018325 |
| *Rhodothermaceae* | 0.2249±0.0214a | 0.138±0.0134b | 0.003968 |
| *Saprospiraceae* | 0.2007±0.0159a | 0.1186±0.0271b | 0.010597 |
| *Phaselicystidaceae* | 0.1858±0.0218a | 0.1125±0.0179b | 0.010746 |
| *Solimonadaceae* | 0.1336±0.0326b | 0.2127±0.0220a | 0.025241 |

**Table S3** ANOVA for Genus abundance

| Genus | Relative abundance (%) (Mean±SE) | | P-value |
| --- | --- | --- | --- |
|  | BFP3 | CK |  |
| *Halomonas* | 0.7206±0.0692a | 0.0272±0.0152b | 0.000071 |
| *Bacteroides* | 0.3122±0.1583a | 0.0155±0.0113b | 0.031767 |
| *Escherichia-Shigella* | 0.1264±0.0648a | 0.0065±0.0038b | 0.03299 |
| *Delftia* | 0.1071±0.0617a | 0.0057±0.0055b | 0.047093 |
| *Helicobacter* | 0.2293±0.1279a | 0.0131±0.0035b | 0.04288 |
| *Brevibacterium* | 0.1304±0.0538a | 0.0082±0.0030b | 0.017095 |
| *Acinetobacter* | 0.4303±0.2264a | 0.0308±0.0214b | 0.038299 |
| *Photobacterium* | 0.1996±0.0792a | 0.0144±0.0229b | 0.017664 |
| *Lactobacillus* | 0.6011±0.0317a | 0.0948±0.0771b | 0.000463 |
| *Salinimicrobium* | 0.3596±0.1299a | 0.0584±0.0171b | 0.016372 |
| *Truepera* | 0.2529±0.0269a | 0.0509±0.0121b | 0.00029 |
| *Erythrobacter* | 0.1431±0.0355a | 0.0327±0.0046b | 0.005954 |
| *Caulobacter* | 0.2200±0.05460a | 0.0639±0.0297b | 0.012133 |
| *Pseudohongiella* | 0.2495±0.0318a | 0.0787±0.0214b | 0.001515 |
| *Pseudomonas* | 0.3326±0.1003a | 0.1065±0.0057b | 0.017562 |
| *Aquicella* | 0.5824±0.0865a | 0.199±0.0271b | 0.001849 |
| *Flavobacterium* | 0.4334±0.1058a | 0.1542±0.0372b | 0.012523 |
| *Bacillus* | 0.2237±0.0181a | 0.0814±0.0187b | 0.000691 |
| *Niastella* | 0.3076±0.0841a | 0.1236±0.0312b | 0.023719 |
| *Rubrobacter* | 0.1177±0.0087a | 0.0493±0.0112b | 0.001138 |
| *Brevundimonas* | 0.1431±0.0397a | 0.0625±0.0240b | 0.039569 |
| *Arenimonas* | 0.3119±0.0134a | 0.1441±0.0085b | 0.000053 |
| *Pontibacter* | 0.6007±0.0700a | 0.2778±0.0347b | 0.002016 |
| *Nitrosospira* | 0.1235±0.0222aa | 0.0605±0.0135b | 0.013713 |
| *Chthoniobacter* | 0.1064±0.0034a | 0.0566±0.0219b | 0.017766 |
| *Reyranella* | 0.7211±0.0568a | 0.4057±0.0289b | 0.001015 |
| *Mesorhizobium* | 0.334±0.0306a | 0.1966±0.0192b | 0.002744 |
| *Phaselicystis* | 0.1942±0.0226a | 0.1170±0.0187b | 0.010337 |
| *Acidibacter* | 0.5427±0.0897a | 0.3636±0.0311b | 0.030881 |
| *Devosia* | 0.2776±0.0045a | 0.2209±0.0294b | 0.029858 |
| *Gaiella* | 0.5431±0.0119a | 0.4504±0.0289b | 0.006832 |
| *Nitrospira* | 1.1004±0.1509a | 1.4518±0.0564b | 0.019461 |
| *Iamia* | 0.5721±0.0154a | 0.8283±0.0624b | 0.002302 |
| *Aeromicrobium* | 0.1359±0.0172b | 0.2114±0.0059a | 0.001971 |
| *Polycyclovorans* | 0.1397±0.0344b | 0.2213±0.0234a | 0.027332 |

**Table S4** Analysis of COG function differences between in CK and BFP3 soils

| COG | | Treatment | | p-values |
| --- | --- | --- | --- | --- |
| Class1 | Class2 | BFP3 | CK |  |
| POORLY CHARACTERIZED | Function unknown | 7.3400±0.0231 | 7.1885±0.0098 | 0.0249 |
| METABOLISM | Energy production and conversion | 6.3520±0.0251 | 6.4683±0.0047 | 0.0492 |
| METABOLISM | Inorganic ion transport and metabolism | 4.6340±0.0187 | 4.5500±0.0107 | 0.0294 |
| METABOLISM | Amino acid transport and metabolism | 7.9568±0.0082 | 7.8337±0.0054 | 0.004 |
| INFORMATION STORAGE AND PROCESSING | RNA processing and modification | 0.0436±0.0004 | 0.0450±0.0003 | 0.0477 |
| CELLULAR PROCESSES AND SIGNALING | Cell motility | 2.0079±0.0155 | 2.0859±0.0033 | 0.0491 |
| CELLULAR PROCESSES AND SIGNALING | Cell wall/membrane/envelope biogenesis | 6.4947±0.0204 | 6.6308±0.0126 | 0.0342 |
| CELLULAR PROCESSES AND SIGNALING | Signal transduction mechanisms | 6.6542±0.0199 | 6.7703±0.0132 | 0.0323 |
| CELLULAR PROCESSES AND SIGNALING | Extracellular structures | 0.0002±0.00002 | 0.0001±0.000004 | 0.032 |
| CELLULAR PROCESSES AND SIGNALING | Intracellular trafficking, secretion, and vesicular transport | 2.6662±0.0145 | 2.7755±0.0049 | 0.0291 |

Table S5 Alpha diversity of bacterial community in the two samples

| Treatment | Shannon | Simpson | Chao1 | Ace |
| --- | --- | --- | --- | --- |
| CK | 6.4651±0.0105 a | 0.0040±0.0001 a | 1902.4014±13.3676 a | 1881.1497±16.0031 a |
| BFP3 | 6.6321±0.0065 a | 0.0030±0.0001 b | 1877.2804±35.8027 a | 1861.8764±34.5979 a |
